# Single-cell studies of IFN-β promoter activation by wild-type and NS1-defective influenza A viruses

**DOI:** 10.1099/jgv.0.000687

**Published:** 2017-03-20

**Authors:** M. J Killip, D Jackson, M Pérez-Cidoncha, E Fodor, R. E Randall

**Affiliations:** ^1^​ School of Biology, Biomedical Sciences Research Complex, North Haugh, University of St. Andrews, Fife KY16 9ST, UK; ^2^​ Sir William Dunn School of Pathology, University of Oxford, Oxford OX1 3RE, UK

**Keywords:** influenza virus, NS1, interferon, IFN, innate immune response, interferon antagonist

## Abstract

Deletion or truncation of NS1, the principal IFN antagonist of influenza viruses, leads to increased IFN induction during influenza virus infection. We have studied activation of the IFN induction cascade by both wild-type and NS1-defective viruses at the single-cell level using a cell line expressing GFP under the control of the IFN-β promoter and by examining MxA expression. The IFN-β promoter was not activated in all infected cells even during NS1-defective virus infections. Loss of NS1 expression is therefore insufficient *per se* to induce IFN in an infected cell, and factors besides NS1 expression status must dictate whether the IFN response is activated. The IFN response was efficiently stimulated in these cells following infection with other viruses; the differential IFN response we observe with influenza viruses is therefore not cell specific but is likely due to differences in the nature of the infecting virus particles and their subsequent replication.

## Abbreviations

ISG, interferon-stimulated gene; NP, nucleoprotein; PAMP, pathogen-associated molecular pattern; PRR, pathogen recognition receptor; RNP, ribonucleoprotein.

## Full-Text

The IFN arm of the innate immune response restricts virus replication and spread during *in vivo* virus infections prior to activation of the adaptive immune system. Recognition of certain viral molecular structures (pathogen-associated molecular patterns, PAMPs) as non-self by pathogen recognition receptors (PRRs) enables an infected cell to detect the presence of a virus and activate the IFN induction cascade, leading to stimulation of the IFN promoter, IFN expression and secretion. IFN then elicits an ‘antiviral state’ in infected cells or surrounding uninfected cells through the upregulation of hundreds of different interferon-stimulated genes (ISGs) that possess either direct or indirect antiviral activity, in order to efficiently limit further replication and spread of the virus (reviewed in Randall and Goodbourn [1]). For influenza A viruses, a predominant PAMP is believed to be the region of partially double-stranded, 5′-triphosphorylated RNA that forms between the partially complementary termini of the influenza A virus genome segments [2, 3]; stretches of dsRNA directly adjacent to a 5′-triphosphate can function as ligands for the PRR RIG-I, which has been shown to be critical for IFN induction during influenza A virus infections [4]. However, these genome segments do not generally exist as free RNA but are encapsidated by viral nucleoprotein (NP) and polymerase to form ribonucleoproteins (RNPs), which are likely to impact the ability of RIG-I to recognize viral genomes. The precise origin of influenza virus PAMPs during infection is therefore still unclear, and contradictory reports exist on the importance of incoming RNPs and the requirements for viral RNA synthesis for the induction of IFN [5, 6]. Additionally, although RIG-I has long been considered the PRR for influenza virus, MDA5 was recently implicated as performing a more significant role in this process than previously thought [7]. Several distinct PAMPs that stimulate different PRRs could therefore be generated during the course of an influenza virus infection.

Like other viruses, influenza virus encodes factors that antagonize the IFN response in order to be able to replicate efficiently. Although other viral proteins have been reported to modulate IFN expression [8–11], the principal of these is the NS1 protein, which limits IFN expression at several different stages of the IFN induction pathway and can additionally inhibit the expression and/or function of ISG products downstream. As a result, viruses with NS1 deletions or truncations induce large amounts of IFN and are attenuated in IFN-competent systems [12–14]. The RNA-binding domain of NS1 has been implicated in preventing activation of the IFN-β promoter during infection by sequestering dsRNA away from PRRs, and recombinant viruses expressing RNA binding mutants of NS1 induce higher levels of IFN than wild-type (wt) virus [15–18]. NS1 also inhibits RIG-I and downstream IFN induction by targeting the TRIM25 ubiquitin ligase that is required for RIG-I activation [19–22]. Post-transcriptional inhibition of IFN expression by NS1 additionally occurs through the binding and inhibition of the 30 kDa subunit of the cellular cleavage and polyadenylation specificity factor (CPSF30), which prevents the processing of all cellular pre-mRNA 3′-ends, thereby globally limiting the expression of host genes including IFN-β and ISGs [23–26]. The relative contribution of each of these functions towards overall limiting of IFN expression differs between virus strains, since some strains do not target CPSF30 while others are unable to prevent IFN induction upstream of the IFN promoter [26, 27].

**Fig. 1.**
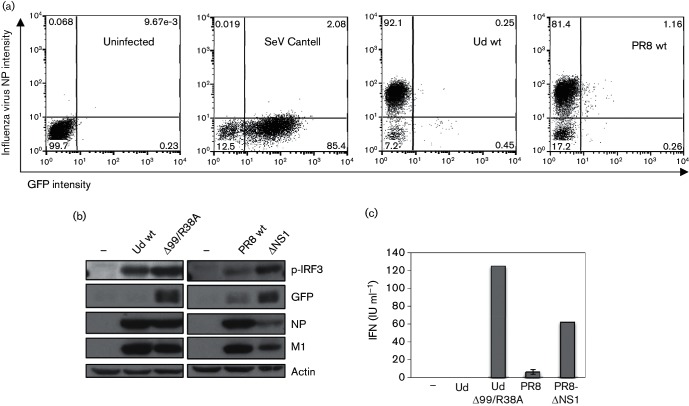

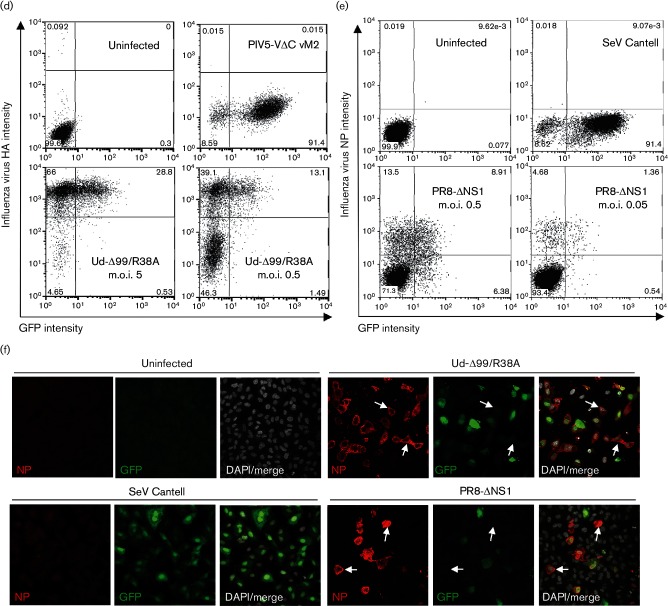
Failure to activate the IFN-β promoter by NS1-defective influenza A viruses. (a) A549/pr(IFN-β).GFP cells were uninfected or infected with Ud wt, PR8 wt or Sendai virus (SeV) Cantell at 5 p.f.u. cell^−1^. At 16 h post-infection (p.i.), cells were trypsinized, fixed, permeabilized and immunostained with antibody against influenza virus NP and subsequently analysed for NP and GFP expression by flow cytometry. Cells were divided into quadrants according to intensity of NP and GFP expression, and the percentage of cells in each quadrant is indicated on each graph. (b) Cell lysates were generated from A549/pr(IFN-β).GFP monolayers infected with Ud wt, Ud-Δ99R38A, PR8 wt or PR8-ΔNS1 at 5 p.f.u. cell^−1^ or uninfected cells for 16 h p.i., then subjected to SDS-PAGE and immunoblotting with antibodies specific to phospho-IRF3, GFP, viral proteins and actin. (c) Cells were treated as in (b). IFN present in culture media was estimated by a cytopathic effect-reduction bioassay [31]. Error bars represent the results of three independent experiments. (d, e) A549/pr(IFN-β).GFP cells were uninfected or infected with Ud-Δ99/R38A or PR8-ΔNS1 at the multiplicities indicated on the plots. At 16 h p.i., cells were trypsinized, fixed, permeabilized and immunostained for influenza virus haemagglutinin or NP expression as indicated. PIV5-VΔC vM2 [32] or SeV Cantell infections were also carried out as positive controls for GFP expression. Cells were analysed by flow cytometry as in (a). (f) Cells were infected as in (d) and (e). At 16 h p.i., cells were fixed, permeabilized and immunostained for influenza virus NP. GFP, NP (red) and nuclei (stained with DAPI; grey) were visualized by confocal microscopy. Arrows denote those cells that are strongly positive for virus antigen but in which GFP cannot be detected.

Most previous studies examining IFN induction by influenza viruses have used methods that give an indication of the average response across a cell population; consequently, little information exists on the activation of innate immune responses to influenza virus infection at the single-cell level. In this study, we have extended our previous work into the examination of IFN induction at the single-cell level by negative-sense RNA viruses [28–30] to study IFN induction by influenza viruses in greater depth. Activation of the IFN induction cascade in individual infected cells was examined using the A549/pr(IFN-β).GFP cell line; these human lung epithelial cells express GFP under the control of the IFN-β promoter and consequently respond very effectively to IFN inducers, including synthetic dsRNA and stocks of paramyxoviruses that are rich in defective viruses (Fig. 1a, b) [28, 30–32]. As we have reported previously [28, 29], GFP expression in cells infected with A/Udorn/72 (Udorn; H3N2) and A/Puerto Rico/8/34 (PR8; H1N1) influenza A viruses was observed only in a very low percentage of infected (i.e. NP-positive) cells (0.25 and 1.16 %, respectively) (Fig. 1a). While this result clearly indicates that the majority of infected cells are negative for IFN-β.GFP expression, this does not necessarily mean that the IFN induction cascade has not been activated upstream of GFP protein expression in these GFP-negative cells. In cells infected with certain strains of influenza virus, the IFN-β promoter can be activated and IFN-β pre-mRNA generated, yet mature IFN-β mRNA is not formed and IFN-β protein is not expressed due to NS1-mediated inhibition of CPSF30 activity [27]. As such, viruses that target CPSF30 (including Udorn wt) prevent expression from both constitutively active and inducible promoters, which has implications for the expression of IFN and ISGs [26]. Consistent with this, considerable IRF3 activation (an indicator of IFN induction upstream of the IFN-β promoter) can be detected by Western blot in Udorn wt-infected A549/pr(IFN-β).GFP cells, yet GFP expression cannot be detected (Fig. 1b). However, this was not the case for PR8 wt-infected cells in which IRF3 activation correlated with GFP expression (Fig. 1b), consistent with the inability of this virus to inhibit CPSF30 [26, 33–35].

**Fig. 2.**
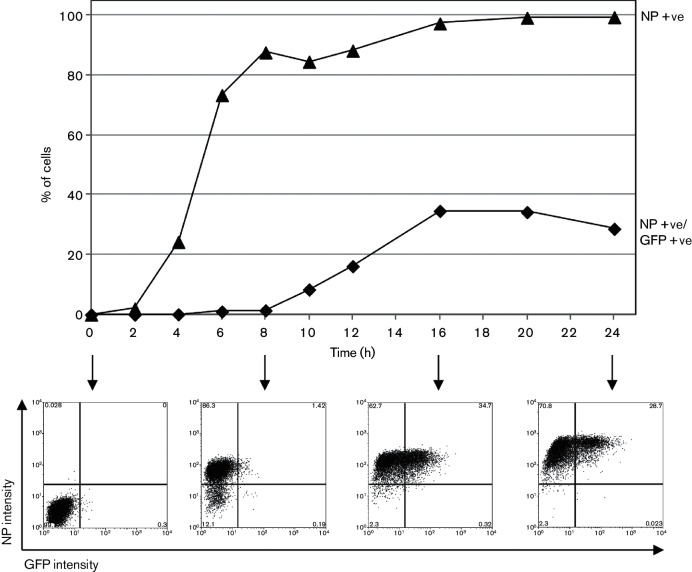
Timecourse of GFP expression in A549/pr(IFN-β).GFP cells during infection with an NS1-defective influenza A virus. A549/pr(IFN-β).GFP cells were infected with Ud-Δ99/R38A at 5 p.f.u. cell^−1^. At the indicated times post-infection, cells were trypsinized, fixed, permeabilized and immunostained for NP expression. GFP and NP expression were subsequently analysed by flow cytometry. The percentage of cells positive for NP and the percentage of cells positive for both NP and GFP at each timepoint are plotted. Flow cytometry plots at selected timepoints are shown below the graph.

Given the well-described role of NS1 in limiting IFN induction, we sought to examine the effect of deleting NS1 on expression of our IFN-β reporter by infecting A549/pr(IFN-β).GFP cells with influenza viruses that lack a functional NS1 protein. Ud-Δ99/R38A, a recombinant Udorn virus, has an R38A mutation in the RNA-binding domain that abrogates the dsRNA-binding activity of the NS1 N terminus [36, 37] and lacks most of the C-terminal effector domain of NS1 [38]. Consequently, Ud-Δ99/R38A also lacks binding sites for TRIM25 and CPSF30 [19, 23, 34], and IRF3 activation in cells infected with Ud-Δ99/R38A therefore correlates well with GFP expression, in contrast to cells infected with the parental Udorn wt virus (Fig. 1b). PR8-ΔNS1 has a complete NS1 gene deletion in the PR8 background [12]. Consistent with a loss of IFN-antagonist activity, both Ud-Δ99/R38A and PR8-ΔNS1 induce considerably more IFN secretion from infected cell monolayers than their respective wt viruses (Fig. 1c). When activation of the IFN induction cascade by these viruses was examined by flow cytometry, infection with Ud-Δ99/R38A and PR8-ΔNS1 resulted in a higher number of GFP-positive infected cells (Fig. 1d, e) than seen for Ud wt or PR8 wt (Fig. 1a), due to alleviation of the NS1-mediated inhibition of IFN expression that exists during wt virus infections. However, strikingly, a considerable number of infected cells that were strongly positive for viral protein remained negative for GFP, indicating that the IFN induction cascade had not been activated despite viral replication occurring in these cells. In contrast, our positive controls in these experiments, paramyxovirus preparations rich in defective viruses (including the Cantell preparation of Sendai virus, which like influenza virus is known to generate RIG-I ligands [2, 39]), induced GFP in the majority of infected cells (Fig. 1a, d, f). In support of the flow cytometry data (Fig. 1d,f), Ud-Δ99/R38A and PR8-ΔNS1-infected cells that are positive for viral protein but negative for GFP expression were also clearly seen by microscopy (Fig. 1f). This pattern of IFN-β promoter activation was not due to examination of GFP expression too early or too late in infection since it was observed over a time course of Ud-Δ99/R38A infection: by 8 h p.i., the majority of cells were positive for viral NP and GFP-positive cells could be detected in small numbers (Fig. 2). The number of GFP-positive cells peaked at 16 h p.i. and remained stable until 24 h, when cell death led to a slight drop in GFP expression due to the apoptogenic nature of the Δ99 NS1 deletion [38].

**Fig. 3.**
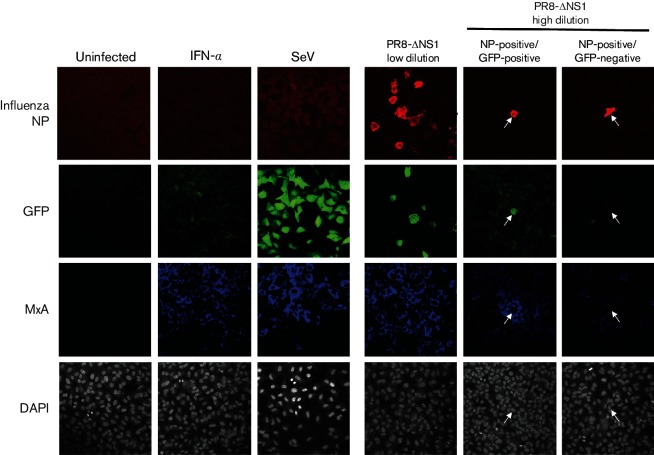
Heterogeneity in the induction of an antiviral state in uninfected cells surrounding NS1-defective influenza A virus-infected cells. A549/pr(IFN-β).GFP cells were infected with low or high dilutions of PR8-ΔNS1 as indicated, uninfected or infected with SeV Cantell as a positive control for GFP expression. As a positive control for MxA expression, cells were treated with IFN-α (1000 IU ml^−1^). Cells were fixed at 16 h p.i., permeabilized and immunostained for influenza virus NP and MxA. Nuclear material was stained with DAPI. GFP, MxA, NP and DAPI staining were examined by confocal microscopy. Arrows denote the positions of NP-positive cells that are either GFP positive and surrounded by MxA-positive cells or are GFP negative and surrounded by MxA-negative cells.

We have clearly demonstrated that an IFN-β reporter gene is not expressed in a subpopulation of cells infected with NS1-defective influenza viruses, despite the multitude of ways in which NS1 functions to limit IFN induction during wt virus infections. We next examined expression of an endogenous marker of activation of the IFN response, the ISG product MxA, in A549/pr(IFN-β).GFP monolayers infected with PR8-ΔNS1. Basal MxA expression was low in uninfected cells but was upregulated following treatment with exogenous IFN or infection with SeV Cantell (Fig. 3). At low dilutions of PR8-ΔNS1, in regions of the cell monolayer in which several GFP-positive cells were present, the surrounding uninfected cells were MxA positive; this was consistent with IFN having been secreted from GFP-positive cells and eliciting an antiviral state in neighbouring uninfected cells. At higher dilutions of PR8-ΔNS1, fields of view containing one or two infected cells could be detected since PR8 does not undergo multi-cycle replication in tissue culture unless trypsin is added to the culture media. In fields of view containing NP-positive, GFP-positive cells at a high dilution of virus, surrounding uninfected cells were positive for MxA indicating the establishment of an antiviral state in these cells (Fig. 3: NP-positive/GFP-positive panel). In contrast, uninfected cells surrounding cells that were strongly NP positive but GFP negative were negative for MxA (Fig. 3: NP-positive/GFP-negative panel), strongly suggesting that IFN had not been secreted from these infected cells.

Using an IFN-β reporter gene system and endogenous MxA expression studies, we have demonstrated that both wt viruses and viruses lacking a functional NS1 protein, which are incapable of efficiently inhibiting IFN production and are robust activators of the IFN response in cell populations, stimulated IFN induction pathways in only a subset of infected cells. Thus, only a subset of infected cells is likely to be responsible for secreting the IFN that is detectable during both wt- and NS1-defective virus infections. A previous study used an IFN-β-luciferase mouse model to study the cell types responsible for IFN secretion in the infected mouse lung, and found that luciferase expression was restricted to relatively few epithelial cells and macrophages that had been infected with both the wt and an NS1-deletion mutant of the mouse-adapted H7N7 SC35M strain [40]. Furthermore, such differential expression has similarly been noted for ISGs, with only about 20 % of cells infected with an NS1-deletion mutant of the A/Panama/2007/1999 strain going on to express ISG15 [41]. In an *in vivo* study by Kalfass and colleagues, differences in the susceptibility of cell types to influenza virus infection or cell-to-cell variability in the ability to mount an IFN response may have contributed to the differential IFN-β promoter activation between cells. In the present study, these potential sources of variability have been eliminated; the subcloned A549/pr(IFN-β).GFP reporter cell line can respond relatively uniformly to IFN inducers (Fig. 1a, d–f) [28, 30, 31]. Our data thus provide evidence that these cells are differentially mounting an IFN response due to differences in the nature of the infecting virus particles and their subsequent replication rather than cell-specific factors. Indeed, we have shown previously that different preparations of the same PR8 virus activate the IFN response to very different degrees [29].

We have demonstrated that influenza viruses that are defective in NS1, the principal IFN antagonist, have the ability to enter cells and replicate without leading to IFN induction. Other viral proteins, such as PB2 or PB1-F2, have been previously reported to inhibit IFN induction [8–11], and we cannot rule out that these proteins are actively inhibiting IFN induction downstream of PAMP recognition in cells infected with an NS1-deficient virus. However, we favour the interpretation that the replication cycle of influenza viruses is such that it is likely the virus can replicate without generating or exposing PAMPs (e.g. by replicating in the nucleus, in a different subcellular compartment to the cytoplasmic PRRs and by efficiently encapsidating the viral RNA genome and its full-length cRNA into RNPs), thereby preventing activation of the IFN response during normal virus replication (reviewed in Killip *et al.* [29]). Furthermore, our data do not support a significant role for incoming genomes in IFN induction, as has been reported previously [6, 42], since the majority of cells infected with NS1-defective viruses do not express markers of IFN response activation despite all cells having been exposed to incoming nucleocapsids. Interestingly, RIG-I has been reported to recognize RNPs from avian influenza viruses more readily than those from human viruses [6]; thus, species-specific differences could exist in the number of cells expressing IFN following infection with NS1-deficient viruses of human or avian origin.

Our results suggest that factors in addition to NS1 expression status determine the IFN activation status of a cell infected with influenza virus, and that the triggering of IFN induction pathways is likely to be associated with some form of aberrant replication, e.g. inefficient genome encapsidation, the generation of aberrant RNA products or the replication of defective genomes. There is accumulating evidence pointing to the involvement of the latter in this process [2, 29, 43–48], and a link between defective genomes and IFN induction is well documented for other negative-sense RNA virus families. Thus, rather than being required to limit IFN production in cells in which virus is replicating normally, the primary function of NS1 may be to limit IFN induction in the event of these aberrant replication events occurring.
